# An Undergraduates' Hospital

**Published:** 1905-11-25

**Authors:** 


### AN UNDERGRADUATES' HOSPITAL.

### THE STILLMAN INFIRMARY, CAMBRIDGE, MASS.

This institution, which was recently built, has an attractive exterior and stands in an open situation, not far from Harvard College. The matron, Miss Dark, is a graduate of the Massachusetts General Hospital Training School, and is evidently the right woman in the right place. She had everything in good order. Indeed the whole hospital, together with the infectious pavilion, was beautifully kept and most attractive and comfortable in appearance. This institution is hygienically up to date. The wards are airy and well lighted, the single rooms well planned and efficient, and the whole of the adjuncts to both, as well as the fittings, are of the most modern and approved type. The sanatorium proper provides twenty-eight beds, and the larger wards contain eight beds each. The infectious pavilion has accommodation for some sixteen patients in addition. The main building of the sanatorium consists of four floors. The ground and the first floor contain the wards. The second floor provides accommodation for the staff, and the kitchens and other offices and store-rooms are placed in the basement. Everything throughout this hospital is well up to date and its equipment is so complete as to exhibit the expenditure of considerable thought, care and knowledge on the part of those responsible. It is interesting to note that the floor of the corridors was made of asbestolith, a similar material to that employed for the floors of the rebuilt Home Hospital, Fitzroy House, Fitzroy Square, London. We could not find any cracks in the asbestolith as used at the Stillman Infirmary, though Miss Dark mentioned that it had given in places. There is in America a similar form of flooring, known as the "Crown," which is stated to be the best of this type of construction; but we did not come across any institution in which it had actually been used for hospital floors. The flooring in the wards was hard rift pine. "Rift" is used in America to indicate that the wood has been quartered so as to present the hardest surface when it is laid down as flooring. Miss Dark is evidently a good and wise disciplinarian. If anybody doubts the fact they had better study the provision she has made for the use of those students who are smokers. This consists of a small glass surrounded lean-to outside the buildings proper, which on the day of our visit contained two chairs, one of which was attractive looking and the other was not.

Every student registered in any Cambridge department of the Harvard University is charged a fee of four dollars (sixteen shillings) a year for the maintenance of the Stillman Infirmary. This payment entitles every student, on the order of a physician, in case of sickness, to receive in return a bed in a ward, board, and ordinary nursing for a period not exceeding two weeks in any one academic year. Students in departments outside Cambridge may obtain the same privileges at the Infirmary, by a voluntary payment of the same fee of four dollars, on or before October 10, in each academic year. Unmarried officers of the University, in case of sickness, provided they have paid the annual fee of four dollars, are given similar privileges.

The Stillman Infirmary has funds amounting to about $61,000 (£12,400), the income from which is devoted to its maintenance. This income, together with the general fees just stated, will, it is estimated, meet the expenses of the institution for the present. The object with which the Stillman Infirmary was founded, and its income provided, is, to cause sick students to make prompt application to the Infirmary, where they can be taken care of in the best manner, instead of remaining in their rooms, where it is difficult to secure that they shall be well cared for. Altogether, the Stillman Infirmary may be regarded as a good type of the University sanatorium, and it would be well if the president and senate of each of our great Universities were to take steps to secure the provision of similar accommodation.

